# The personalization paradox: how AI-driven adaptive learning environments are associated with college students’ academic emotions and self-regulation learning—a moderated mediation model

**DOI:** 10.3389/fpsyg.2026.1915839

**Published:** 2026-09-11

**Authors:** Jing Li, Ziheng Lin, Chengqun Qiu

**Affiliations:** 1 School of Physics and Electronic Engineering, Yancheng Teachers University, Yancheng, China; 2 Graduate School of Zhejiang University, Hangzhou, China; 3 Innovation Entrepreneurship College, Yancheng Teachers University, Yancheng, China

**Keywords:** academic emotions, AI literacy, AI-adaptive learning environment, control-value theory, longitudinal design, moderated mediation, personalization paradox, self-regulated learning (SRL)

## Abstract

**Background:**

Artificial intelligence (AI)-engaged adaptive studying platforms are being rapidly adopted across higher education. While they provide significant opportunities to tailor students with both educational content and learning paces to individual needs, educators and researchers proposed their concerns that over-reliance on algorithmic itself may unconsciously be negatively associated with students’ ability in self-regulated learning (SRL)—a phenomenon termed the “personalization paradox.”

**Aims:**

Integrating control-value theory (CVT) and self-regulated learning (SRL) theory, this study demonstrates that academic emotions—enjoyment, anxiety, and boredom—mediate the relationship between perceived AI-adaptive environments and SRL, with CVT clarifying why these environments foster emotional responses and SRL theory explaining how such emotions translate into behavioral outcomes. This study uniquely combines CVT and SRL within a single moderated mediation structure, providing an innovative theoretical lens to know the personalization paradox very well.

**Methods:**

A three-wave longitudinal survey was applied across one semester during the academic year with 486 undergraduates from four universities in China. At T1, students reported their perceptions of the AI-adaptive surroundings and AI literacy to researchers; at T2, they reported academic emotions; at T3, they reported their SRL behaviors. Structural equation modeling with latent interaction terms and cluster-robust standard errors were utilized to estimate the moderated mediation structure.

**Results:**

Perceived AI-adaptive environments were negatively related to SRL (*β* = −0.19, *p* < 0.01), giving certain supports to personalization paradox. Indirect effects in enjoyment (*β* = −0.08), anxiety (*β* = −0.06), and boredom (*β* = −0.09) were completely significant, respectively accounting for 54.8% of the entire effect. Most importantly, AI literacy moderated the first selective pathways from the AI environment to every academic emotion (e.g., enjoyment was *β* = 0.14, *p* < 0.01). such that the negative emotional impacts were moderated among students with higher AI literacy. This model accounted for 28.4% of the variance in SRL.

**Conclusion:**

The personalization paradox operates through affective pathways, with AI literacy playing the role as a significant protective index. These results highlight the emotional and autonomy-related trade-offs inherent in algorithmic personalization and suggest that universities embed AI literacy as a credit-bearing module and design adaptive systems with lucid, user-controllable recommended interfaces. Causal inferences still are limited given the existing observational design.

## 1 Introduction

The landscape of higher education is undergoing a rapid transformation driven by artificial intelligence, with marked increases in intelligent tutoring systems, adaptive educational platforms, personalized learning pathways, as well as real-time learning analytics (Holmes et al., 2019; Zawacki-Richter et al., 2019). These techniques continuously calibrate learning content complexity, content sequencing, and corresponding feedback in accordance with the learner’s deeds, behaviors and performance (Pane et al., 2017; VanLehn, 2011). Personalization basically institutes the core value view of AI utilization on education (Luckin et al., 2016).

However, scholars have begun to clarify a troubling contradiction: as educational tools become increasingly personalized, learners’ autonomy and self-regulatory skills may be adversely affected (Koedinger et al., 2023; Molenaar, 2022). Unlike user-driven customization, algorithm-driven personalization may shift decision-making authority from learners to systems, thereby influencing users’ perceived agency and control over the learning process (Sundar and Marathe, 2010). When algorithmic systems predict needs and regulate following learning sequences, students have sparse opportunities to independently strategize, tutor and refine their study content. This algorithmic delegation risks conditioning learners as passive receivers rather than active creators of knowledge (Bulger, 2016; Selwyn, 2022). Moreover, hyper-personalized and gamified interfaces have been criticized for undermining intrinsic motivation and autonomy, further fostering passive reliance and lessened perceptive investment (Kulik and Fletcher, 2016; Watters et al., 2024). Researchers have termed this kind of complex phenomenon the “personalization paradox” (Renz et al., 2020).

Despite daily growing theoretical attention, empirical research delving into its intrinsic psychological mechanics underlying this paradox remains sparse. Three vital questions persist: (a) How does AI-adaptive studying environment have relation with SRL? (b) What kind of roles do academic emotions play in such relationship? And (c) what individual learner characteristics might buffer these effects? Answering these questions is necessary for understanding the psychological branches of employing AI in education and for refining the relevant design of educational technologies (Azevedo and Gašević, 2019; Winne, 2022).

To fill this gap and bridge it, the present study integrates the control-value theory (Pekrun, 2006) with the self-regulated learning (SRL) concept (Zimmerman, 2000) to establish a comprehensive moderated mediation model. The researchers specifically investigate how enjoyment, anxiety, and boredom mediate the core relationship, while regarding AI literacy as the underlying moderating variable. The researchers’ principal objective is to illuminate the concealed emotional and autonomy costs of AI-driven personalization, therefore providing contributions through psychological perspectives necessary for the advancing human-originated AI designs in education. Despite growing theoretical communications, no empirical study to our perception has simultaneously (a) examined academic emotions as mediators between AI-adaptive environments and SRL, (b) researched AI literacy as a moderator of these emotional pathways, and (c) employed a longitudinal design to acquire the progressive character of autonomy erosion. The current study emphasize these interlinked gaps.

## 2 Theoretical framework and literature review

### 2.1 AI-adaptive learning environments and the personalization paradox

An AI-adaptive learning environment is defined as a digital ecosystem that leverages AI to actively calibrate educational content, instructional routes, pacing, and feedback based on real-time evaluations of a learner’s knowledge foundation, behavioral trends, sensing state, or emotional temperament (Baker, 2016; Shute and Rahimi, 2017). While adaptivity facilitates personalization, it may at the same time trigger unintended psychological consequences. The delegation of decision-making to algorithms can tremendously reduce the meaningful choices learners typically make—such as deciding what studying material to learn, what to do with it, and the best methodologies to employ—therefore limiting their chances to exercise and optimize their metacognitive skills (Azevedo et al., 2022; Glogger-Frey et al., 2022; Molenaar et al., 2023). This phenomenon of delegating cognitive responsibilities onto machines has been connected to a potential decline in metacognitive abilities (Clark and Mayer, 2023; Dillenbourg, 2013).

From the perspective of self-determination theory (Deci and Ryan, 2000), adaptive platforms might enhance competence through prompt and aimed feedback yet, they simultaneously risk undermining autonomy if the learning process becomes overly algorithms-reliance. A diminished sense of ownership over one’s educational trip is known to degrade intrinsic motivation and deep cognitive engagement (Ryan and Deci, 2017). Consequently, the personalization paradox quickly captures the inherent disagreement between the immediate gains in learning efficiency and the long-term cultivation of independent, self-motivated learners (Selwyn, 2022; Watters et al., 2024).

### 2.2 Control-value theory of academic emotions

Academic emotions refer to the following affective responses directly tied to achievement-oriented learning activities and their following outcomes (Pekrun, 2006). Control-value theory posits that these emotional learning journeys are basically driven by two main cognitive appraisals: the degree of control a learner perceives over the academic events and results, and the value they ascribe to those events (Pekrun and Perry, 2014). Environments that facilitate high perceived control and high value are keen to introduce positive, activating emotions, such as enjoyment, hope, and pride. Conversely, deficits in either control or value evaluations might be likely to trigger negative emotions, such as disappointment, anxiety, hopelessness and boredom (Pekrun et al., 2011).

In AI-adaptive environments, the traditional pathway of control shifts from “active agency” exercised by the student to a state of “passive reception,” as massive instructional decisions are automated (Huang et al., 2023; Tschofen and Mackness, 2012). This structural change is capable of stimulating distinct emotional responses. Moreover, if algorithmically created fails to align with the learners’ personal interests, their perceived task value might likewise deteriorate (Pekrun and Linnenbrink-Garcia, 2012). Emerging literature supports the applicability of CVT to AI-enhanced educational settings, emphasizing the vital need of fostering learners’ perceived operation and task value (Li et al., 2025; Wang et al., 2025).

### 2.3 Self-regulated learning (SRL) theory

Self-regulated learning (SRL) is defined as an iterative, cyclical process in which learners actively set objectives, monitor their progress, modify their systematized strategies, and engage in reflective evaluation of their performances (Zimmerman, 2000).

This process is typically organized into three phases: forethought (including goal setting and strategic planning), performance (volitional control and strategy implementation), and self-reflection (containing evaluation and attribution). Proficiency in SRL is recognized as a cornerstone to academic achievement and lifelong continuing learning (Pintrich, 2004; Schunk and Greene, 2018).

Paradoxically, the integration of AI-adaptive systems might inadvertently erode these essential skills by outsourcing the vital tasks of planning, monitoring, and strategic regulation to comprehensive algorithms (Azevedo et al., 2022; Molenaar, 2022). The decline is insidious since students often perceive that their learning process becomes “easier” or more fluent, remaining oblivious to the construction by themselves or the algorithms within self-regulated proficiencies (Winne, 2022; Zhao et al., 2024).

The integration of CVT and SRL within our model is theoretically grounded as follows: CVT clarifies why adaptive environments foster emotional responses by altered control-value appraisals; SRL theory demonstrates how these emotional states translates into weakened self-regulatory actions.

Specifically, reduced enjoyment and increased anxiety or boredom impair the forethought and self-reflection application of SRL (Zimmerman, 2000), therefore, undermining the periodical self-regulatory process.

### 2.4 AI literacy as a protective factor

The concept of AI literacy encompasses the foundational knowledge, practical skills, and critical thinking abilities necessary for effective interaction with AI technologies (Long and Magerko, 2020; Ng et al., 2021). As AI gradually becomes increasingly ubiquitous in educational fields, AI literacy is emerging as a critical competency that can buffer the psychological consequences associated with such AI systems (Kong et al., 2024; Wang et al., 2025). Students with higher levels of AI literacy are commonly better positioned to comprehend the underlying logic and limitation of algorithmic systems, enabling them to maintain better agency and engage vitally with AI-generated content (Cetindamar et al., 2024; Ng et al., 2021). Accordingly, robust AI literacy might lessen the negative emotional feedbacks evoked by adaptive environments, thereby alleviating the personalization paradox.

### 2.5 The present study: hypotheses and conceptual model

Integrating the theoretical perspectives outlined above, this study proposed the following hypotheses:

*H1*: Perceived AI-adaptive learning environment will be negatively related to students’ self-regulated learning (SRL), reflecting the personalization paradox phenomenon.

*H2*: Academic emotions—specifically, enjoyment, anxiety, and boredom—will mediate the association between the perceived AI-adaptive environment and self-regulated learning (SRL). This yields three sub-hypotheses:

*H2a*: Enjoyment plays a mediating role in such a relationship.

*H2b*: Anxiety plays mediating role in such a relationship.

*H2c*: Boredom plays a mediating role in such a relationship.

*H3*: AI literacy will moderate the initial pathways connecting the AI-adaptive environment to academic emotions. Specifically, higher AI literacy is supposed to eliminate the negative emotional effects associated with these environments.

Theoretically, AI literacy is defined as a resource that forms learners’ immediate evaluation of AI systems (control-value appraisals) rather than their downstream self-regulatory behaviors directly. That is to say, AI literacy enables learners to interpret and vitally evaluate algorithmic suggestions as they meet them, thereby moderating the primary emotional response to the adaptive environment. It need not moderate the emotion-SRL link because as long as an emotion is experienced, its impact on SRL is majorly automatic and less amenable to knowledgeable resources as AI literacy (Pekrun, 2006).”

**Figure 1:**
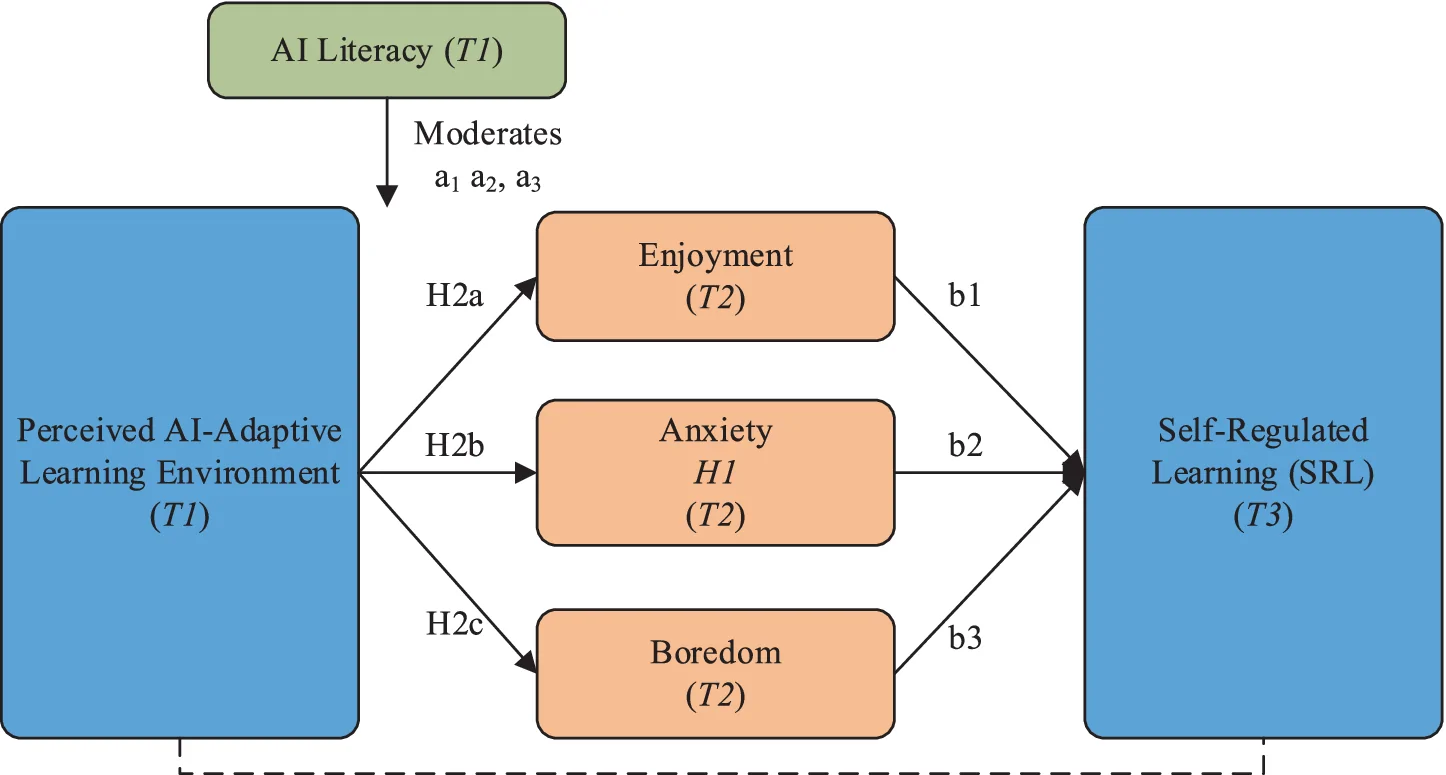
Conceptual moderated mediation model.

The conceptual framework posits this research hypothesizes a moderated mediation structure: AI-adaptive environment → academic emotions (enjoyment, anxiety, boredom) → SRL, in which AI literacy plays as a moderator affecting the first stage of these mediational chains (refer to Figure 1).

## 3 Methods

### 3.1 Participants and procedure

**Figure 2:**
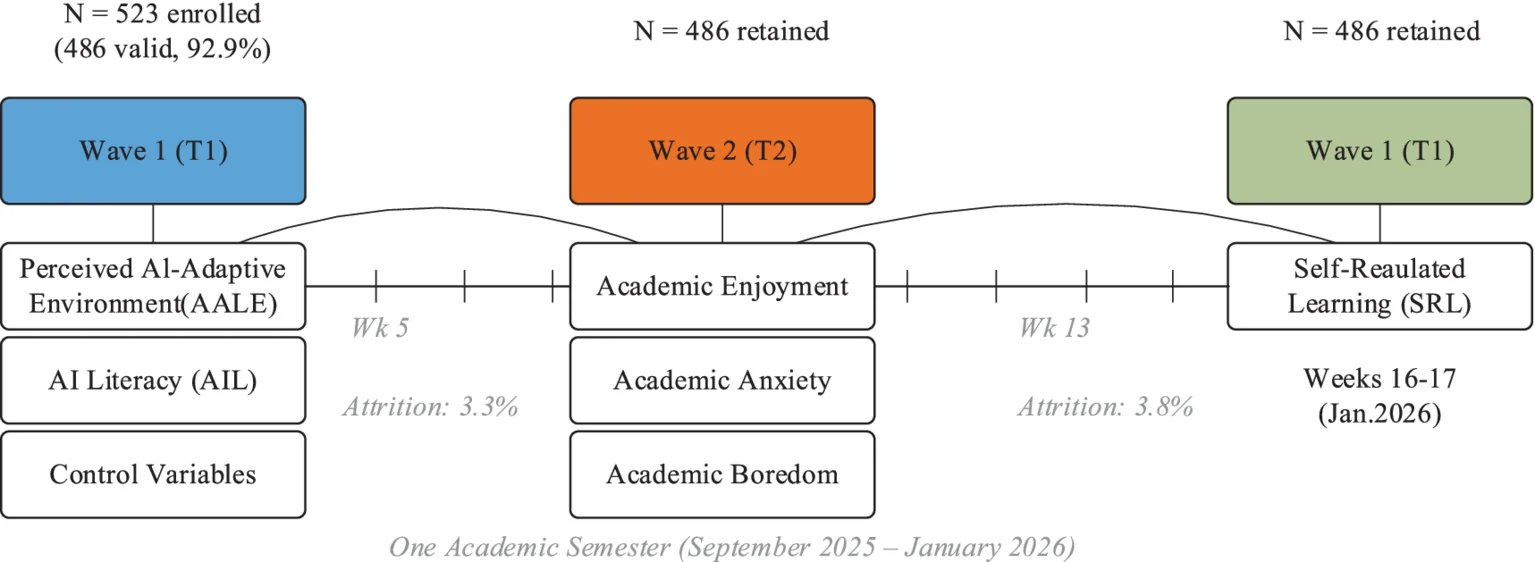
Research design.

To capture the dynamic yet progressive nature of autonomy erosion—a whole process that steps forward progressively over time—we employed a three-wave longitudinal research design, which overcomes the limitations of cross-sectional designs since they are insufficient to achieve our objectives (Little, 2013). Data collection occurred from September 2025 to January 2026. The three waves scheduled at the beginning of the semester (Weeks 2–3, T1), the mid-semester period (Weeks 9–10, T2), and the end of the semester (Weeks 16–17, T3), as shown in Figure 2.

**Table:** 

Time Point	Timing	Variables Measured
T1	Weeks 2–3	Perceived AI-adaptive environment, AI literacy, demographics, AI usage patterns
T2	Weeks 9–10	Academic emotions (enjoyment, anxiety, boredom)
T3	Weeks 16–17	Self-regulated learning (SRL)

The participant pool comprised undergraduate students coming from four universities in China—two comprehensive universities and two research institutions in science and engineering. We adopted a stratified cluster sampling approach, stratifying by university type and academic discipline as the strata, and each individual class as the clusters. A total of 28 classes were selected and recruited: among them, eight from University A (comprehensive, *n* = 142), seven from University B (comprehensive, *n* = 124), seven from University C (science and engineering, *n* = 118), and the remaining six from University D (researching in science and engineering, *n* = 104). Eligibility required all participants to be currently enrolled undergraduates who had engaged with at least one AI-based educational tool during the previous semester and who provided written informed consent.

During the initial wave (T1), we recorded the features of participants’ AI tool usage. The most commonly used tools were general-purpose conversational AI agents, such as ChatGPT and Wenxin Yiyan (52.6%), followed by intelligent tutoring platforms integrated with university systems (34.2%), personalized recommended algorithms embedded in detailed specific courses (28.4%), and AI-driven language learning applications such as Duolingo (18.5%). Only the intelligent tutoring systems and course-specific recommendation tools provided true adaptivity.

To explain this variability, tool type emphasized in supplementary relevant analyses. At the mean value, students noted using these tools for 3.82 h per week (SD = 2.14), mainly for course guidance (58.4%), homework assistance (52.1%), exam preparation (40.7%), and self-initiated study (33.9%).

The initial data selection and collection at T1 gained 523 questionnaires. With the corresponding removal of incomplete submissions, 486 valid answering sheets were retained (retention rate was 92.9%). Attrition was 3.3% from T1 to T2 and 3.8% from T2 to T3, yielding a total attrition rate of 7.6%. The comparisons between retained participants and those who withdrew revealed no significant baseline differences in gender, academic year, or AI literacy, indicating that attrition appears to be random.

**Figure 3:**
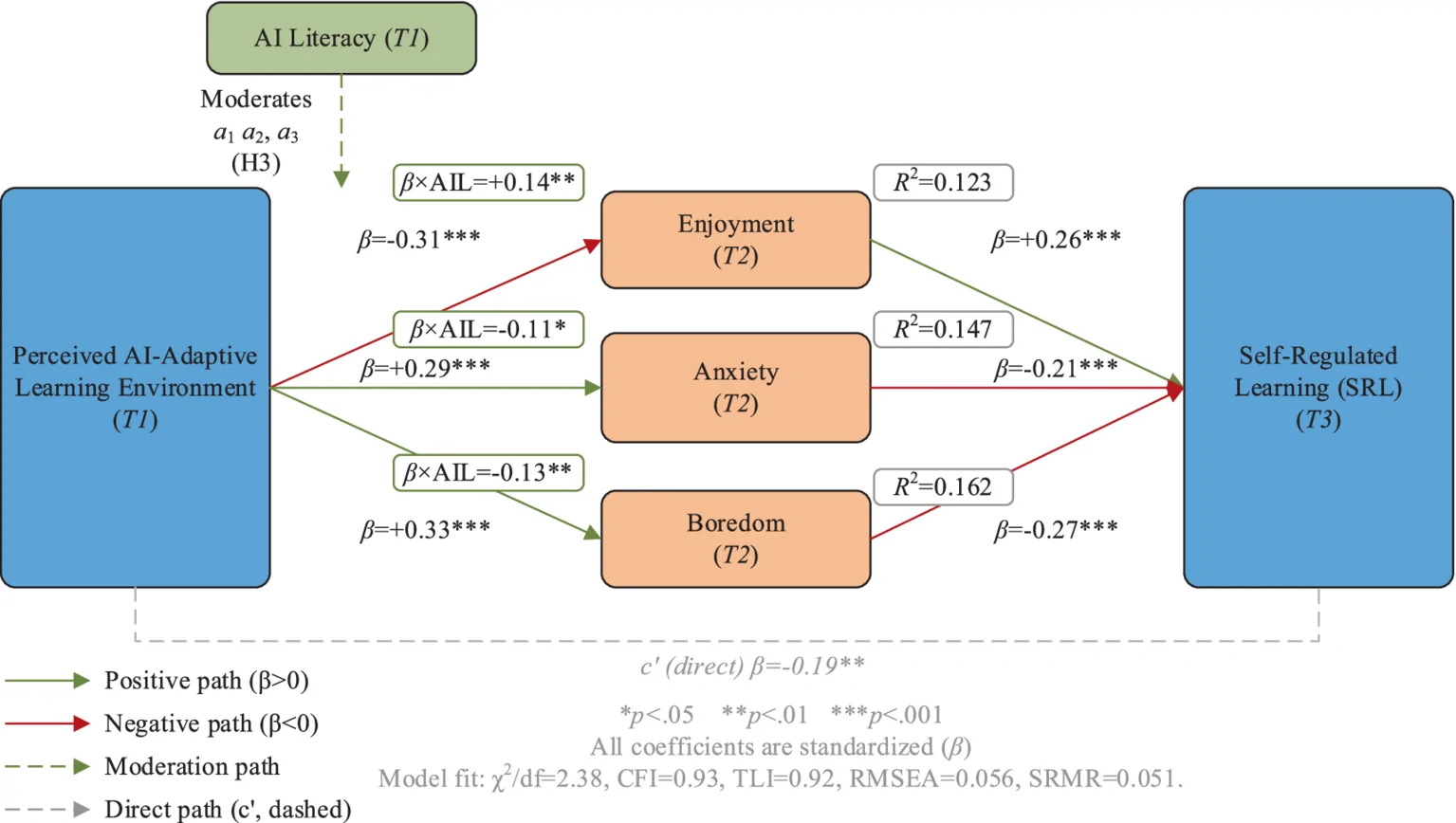
Structural equation model with standardized path coefficient.

The final analytic sample comprised 258 males (53.1%) and 228 females (46.9%), spanning all four academic years. Mean age was 20.47 years (SD = 1.38). Among the tested participants, there were 44.0% Science and engineering students, while 56.0% majoring humanities or social science. *A priori* power analysis via Monte Carlo simulation indicated that 450 participants would be sufficient to detect minor-to-moderate effects with power ≥ 0.80 (Fritz and MacKinnon, 2007); the research’s final sample of 486 clearly exceeded such requirement, refer to Figure 3.

### 3.2 Measures

#### 3.2.1 AI adaptive learning environment perception scale

A novel method was employed grounded in the Technology Acceptance Model (Davis, 1989) and key characteristics of adaptive studying systems (Alkhatlan and Kalita, 2019; Kabudi et al., 2021). The initial item pool covered three dimensions: system adaptability (6 items), degree of personalization (5 items), and algorithmic transparency (4 items). Following content’s validity review by three pedagogical psychology experts, 15 items were reserved and refined by a pilot study. Responseswere recorded on a 5-point Likert scale. The overall scale demonstrated very strong internal consistency (Cronbach’s *α* = 0.89), and second-order CFA gained support on the higher-order structure (χ^2^/df = 2.28, CFI = 0.95, RMSEA = 0.050). The aggregate score was used in primary analyses.

#### 3.2.2 Academic emotions scale

The Chinese amendment edition of the Achievement Emotions Questionnaire (AEQ; Pekrun et al., 2011; Dong et al., 2018) was used to measure the three specific emotions: enjoyment, anxiety, and boredom, with each question five items. The scale leveraged a 5-point Likert format and showed robust reliability (Cronbach’s α: enjoyment = 0.87, anxiety = 0.85, boredom = 0.88).

#### 3.2.3 Self- regulated learning scale

Self-regulated learning (SRL) was evaluated by using a self-report amendment edition of the Chinese version of the Self-regulated learning (SRL) Interview Schedule (SRLIS; Zimmerman and Martinez-Pons, 1986). This 22-item questionnaire covers six domains: aim setting, environmental structuring, task strategies, time handling, help-finding, and self-evaluation. A second-order CFA confirmed that these six dimensions loaded onto a single overarching SRL factor (χ^2^/df = 2.41, CFI = 0.93, RMSEA = 0.055). The scale demonstrated extraordinary reliability (Cronbach’s *α* = 0.91).

#### 3.2.4 AI literacy scale

Drawing on the frame works by Long and Magerko (2020) and Ng et al. (2021), AI literacy was achieved across three dimensions: AI knowledge, AI skills, and AI critical thinking, with four items of each dimension. The 5-point Likert scale showed good internal consistency (Cronbach’s α = 0.88), and a second-order CFA validated the existence of a single higher-order factor.

#### 3.2.5 Control variables

Analyses controlled for gender, academic year, discipline type (science/engineering vs. humanities/social sciences), weekly hours of AI tool use, and the primary type of AI tool used.

### 3.3 Data collection

At each wave, well trained research assistants handled paper-and-pencil questionnaires during regular class sessions Participants were informed of the voluntary nature of this study, confidentiality protections, and their right to withdraw without penalty. Unique personal identification codes were used to link responses across the three waves. Ethical approval was obtained from the Institutional Review Board [IRB-2025-042, August 15, 2025], and all the adult participants provided written informed consent.

### 3.4 Data analysis strategy

Data was analyzed by using SPSS 26 and Mplus 8.3. The analytical order proceeded as below: (1) calculation of descriptive statistics and bivariate correlations; (2) common method bias via Harman’s single-factor test and a marker-variable approach; (3) the measurement model was validated through confirmatory factor analysis (CFA); (4)structural equation modeling (SEM) was used to estimate direct and mediation effects; (5) potential interaction modeling by using XWITH command in Mplus to get moderation; and (6) bootstrapping with 5,000 resamples was used to calculate indirect effects and moderated mediation indices. To account for the nested structure of the data (students within classes), cluster-robust standard errors were applied throughout. All continuous variables were mean-centered prior to generating interaction items, and the robust maximum likelihood (MLR) estimator was used. Missing data, which were no more than 5% per variable, were handled using full-information maximum likelihood (FIML). All reported coefficients are standardized unless otherwise noted.

## 4 Results

### 4.1 Common method Bias

Harman’s single-factor test showed that the main factor accounted for only 24.67% of the total variance, well below the commonly mentioned 40% threshold. A supplementary marker-variable analysis using general self-efficacy (Chen et al., 2001) as the marker indicated that this factor explained only 1.3% of variable variance, and the structural coefficients still emerged stable on its inclusion (ΔCFI < 0.01). Together, these results indicate that common method bias does not become a meaningful threat to the validity of the findings.

### 4.2 Descriptive statistics and correlations

The descriptive statistics and correlations matrix (Table 1) were consistent with theoretical expectations. Perceived AI-adaptive environment showed a significant negative correlation with SRL (*r* = −0.33, *p* < 0.01) and with enjoyment (*r* = −0.28),and positive correlations with anxiety (*r* = 0.31) and boredom (*r* = 0.35). Correlations between academic emotions and SRL were also consistent with the proposed model.

**Table 1:** Descriptive statistics and bivariate correlations (*N* = 486).

Variable	M	SD	1	2	3	4	5
1. AI-adaptive environment	3.52	0.78	1.00				
2. Enjoyment	3.38	0.85	−0.28**	1.00			
3. Anxiety	3.15	0.92	0.31**	−0.42**	1.00		
4. Boredom	2.87	0.95	0.35**	−0.51**	0.44**	1.00	
5. Self-regulated learning	3.41	0.72	−0.33**	0.48**	−0.39**	−0.46**	1.00
6. AI literacy	3.28	0.81	0.18**	0.22**	−0.15**	−0.19**	0.31**

**p < 0.01.

### 4.3 Measurement model

The six-factor measurement model—comprising AI-adaptive environment, enjoyment, anxiety, boredom, SRL, and AI literacy—demonstrated good fit to the data (χ^2^/df = 2.38, CFI = 0.94, TLI = 0.93, RMSEA = 0.052, SRMR = 0.047). All standardized factor loadings were statistically significant, ranging from 0.52 to 0.81. Composite reliability and average variance extracted (AVE) exceeded the recommended thresholds of 0.70 and 0.50, respectively, and the square root of each AVE exceeded the inter-construct correlations, confirming discriminant validity. The six-factor solution fitted significantly better than all tested alternative models (see Table 2).

**Table 2:** Discriminant validity matrix table (Fornell–Larcker criterion).

Construct	1	2	3	4	5	6
1. AI-Adaptive Environment	**(0.79)**					
2. Enjoyment	−0.28	**(0.82)**				
3. Anxiety	0.31	−0.42	**(0.80)**			
4. Boredom	0.35	−0.51	0.44	**(0.81)**		
5. SRL	−0.33	0.48	−0.39	−0.46	**(0.76)**	
6. AI Literacy	0.18	0.22	−0.15	−0.19	0.31	**(0.78)**

The square root of AVE is in the diagonal brackets, and the correlation coefficient is below the diagonal. All AVE square roots are greater than the correlation coefficient, supporting discriminative validity.

### 4.4 Direct and mediation effects

**Figure 4:**
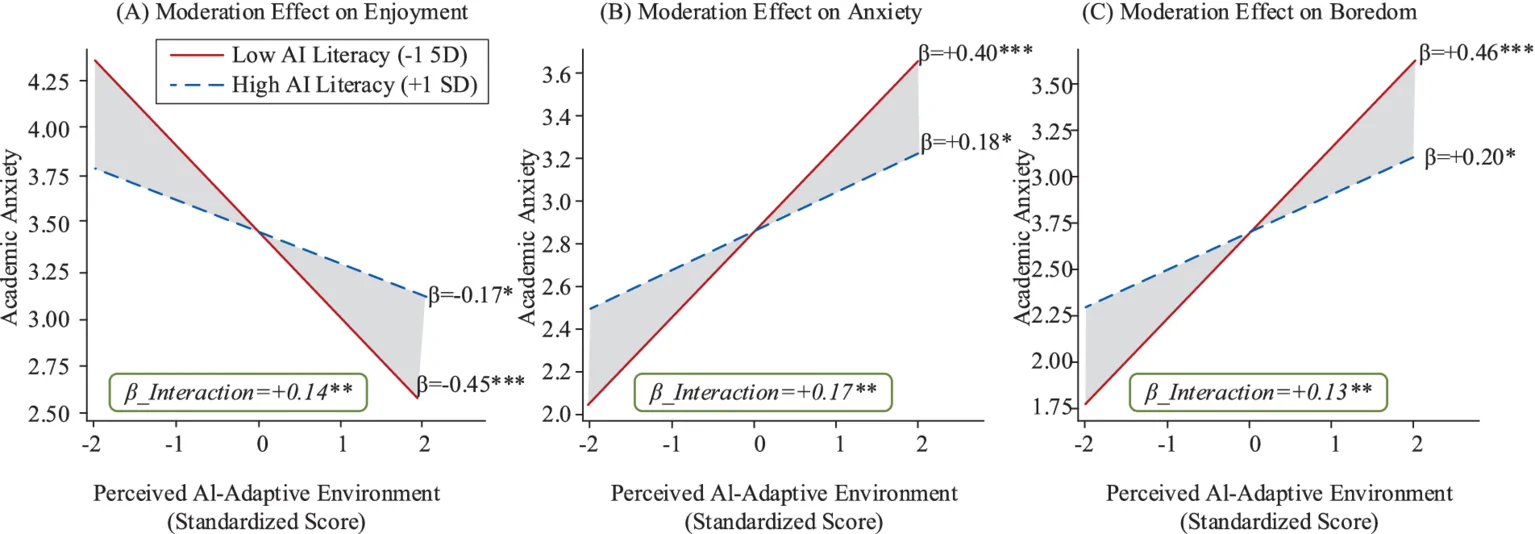
Moderating effect of AI literacy on the relationship between AI-adaptive environment and academic emotions.

The full structural model, including the control variables, showed a satisfactory fit (χ^2^/df = 2.38, CFI = 0.93, TLI = 0.92, RMSEA = 0.056, SRMR = 0.051). Supporting Hypothesis 1, the AI-adaptive environment exhibited a significant negative direct effect on SRL (*β* = −0.19, SE = 0.06, *p* < 0.01, 95% CI [−0.31, −0.07]). The overall model explained 28.4% of the variance in SRL (*R*^2^ = 0.284), as well as 12.3% in enjoyment, 14.7% in anxiety, and 16.2% in boredom, refer to Figure 4.

The mediation analyses offered significant support for Hypotheses 2a, 2b, and 2c. Significant indirect effects were observed through enjoyment (*β* = −0.08, SE = 0.02, 95% CI [−0.13, −0.04]), anxiety (*β* = −0.06, SE = 0.02, 95% CI [−0.11, −0.02]), and boredom (*β* = −0.09, SE = 0.05, *p* < 0.001, 95% CI [−0.15, −0.05]). The total indirect effect was robust (*β* = −0.23, SE = 0.05, *p* < 0.001, 95% CI [−0.33, −0.13]), accounting for 54.8% of the total effect (*β* = −0.42, SE = 0.07, *p* < 0.001, 95% CI [−0.56, −0.28]). Standardized path coefficients were as below: AI-environment → enjoyment (*β* = −0.31, SE = 0.05, *p* < 0.001), enjoyment → SRL(*β* = 0.26, SE = 0.06, *p* < 0.001),; AI-environment → anxiety (*β* = 0.29, SE = 0.05, *p* < 0.001), anxiety → SRL (*β* = −0.21, SE = 0.06, *p* < 0.001); AI-environment → boredom (*β* = 0.33, SE = 0.05, *p* < 0.001), boredom → SRL (*β* = −0.27, SE = 0.06, *p* < 0.001). All hypotheses (H1, H2a, H2b, H2c) were supported.

Given that the direct effect of AI-adaptive environment on SRL remained significant (*β* = −0.19, *p* < 0.01) after accounting for the indirect pathways, these outcomes support partial rather than full mediation, suggesting that while emotional mechanisms massively clarify the personalization paradox, other unmeasured pathways (for example: cognitive or motivational mechanisms) might also contribute.

### 4.5 Moderating effects of AI literacy

To test Hypothesis 3, latent interaction items between the AI environment and AI literacy were specified as predictors of the three academic emotions. The results supported the moderating role of AI literacy across all the three pathways. For enjoyment, the interaction was positive and significant (*β* = 0.14, SE = 0.05, *p* < 0.01, 95% CI [0.04, 0.24]), indicating that higher AI literacy attenuated the negative impact of the environment on this emotion. For anxiety, the interaction was both significant and negative (*β* = −0.11, SE = 0.05, *p* < 0.05, 95% CI [−0.21, −0.01]), showing that AI literacy inhibited the anxiety-inducing effect. For boredom, the interaction was similarly both negative and significant (*β* = −0.13, SE = 0.05, *p* < 0.01, 95% CI [−0.23, −0.03]), indicating that AI literacy reduced the environment’s tendency to foster this emotion.

Simple slope analyses (Table 3) showed that the negative emotional associations were pronounced among students with low AI literacy (−1 SD) but were substantially attenuated for those with high AI literacy (+1 SD). The moderated mediation indices (Table 4) were significant for three pathways, with conditional indirect effects diminishing as AI literacy increased, which fully support Hypothesis 3.

**Table 3:** Simple slopes of AI-adaptive environment → academic emotions by AI literacy level.

Outcome	Low AI literacy (−1 SD)	High AI literacy (+1 SD)
Enjoyment	*β* = −0.38, SE = 0.06, *p* < 0.001	*β* = −0.11, SE = 0.07, *p* = 0.112
Anxiety	*β* = 0.39, SE = 0.06, *p* < 0.001	*β* = 0.15, SE = 0.07, *p* = 0.031
Boredom	*β* = 0.41, SE = 0.06, *p* < 0.001	*β* = 0.16, SE = 0.07, *p* = 0.022

**Table 4:** Moderated mediation indices and conditional indirect effects.

Pathway	Index	95% CI	AI literacy level	Conditional indirect effect	95% CI
Environment → Enjoyment → SRL	0.07	[0.03, 0.12]	Low (−1 SD)	−0.12	[−0.18, −0.06]
			Mean	−0.08	[−0.13, −0.04]
			High (+1 SD)	−0.04	[−0.09, 0.01]
Environment → Anxiety → SRL	0.06	[0.02, 0.10]	Low (−1 SD)	−0.10	[−0.16, −0.04]
			Mean	−0.06	[−0.11, −0.02]
			High (+1 SD)	−0.03	[−0.07, 0.01]
Environment → Boredom → SRL	0.09	[0.05, 0.14]	Low (−1 SD)	−0.14	[−0.20, −0.08]
			Mean	−0.09	[−0.15, −0.05]
			High (+1 SD)	−0.04	[−0.09, 0.01]

All conditional indirect effects and indices are based on 5,000 Bootstrap resamplings with bias-corrected 95% confidence intervals.

## 5 Discussion

### 5.1 The personalization paradox: existence and emotional mechanisms

This study provided robust empirical support for the personalization paradox, demonstrating that students’ perceptions of high adaptive AI learning environments are negatively associated with their self-regulated learning (SRL) capabilities. This finding corroborates theoretical warnings that algorithmic personalization, while efficient, may compromise learner autonomy (Renz et al., 2020; Selwyn, 2022; Watters et al., 2024).

Critically, our research illuminates the emotional mechanisms driving this paradox. The data showed that the AI environment predicted decreased enjoyment and an increased anxiety and boredom, which in turn impaired SRL. The magnitude of these emotional mediations—accounting for more than half of the total effect—suggests that the emotional consequences of AI integration are at least as critical as cognitive ones. The decline in enjoyment likely reflects a loss of autonomy and ownership, consistent with self-determination theory (Ryan and Deci, 2017). Heightened anxiety might stem from reduced perceived control and the unpredictability inherent in algorithmic decision-making (Huang et al., 2023; Tschofen and Mackness, 2012). Boredom, in turn, might arise when algorithmically optimized content lacks sufficient novelty or challenge, thereby obliterating cognitive engagement (Pekrun et al., 2011).

In practical terms, the personalization paradox means the educators and institutions cannot simply assume the AI-driven efficiency unconsciously benefits learning. Specifically, they must be actively monitor and address the emotional side effects of the adaptive AI systems.

### 5.2 The buffering role of AI literacy

The researchers’ moderation analysis highlights AI literacy as a significant protective factor. Students having higher AI literacy experienced tremendously less emotional disruption when engaging with adaptive systems. We propose that this buffering operates through three interrelated mechanisms: (a) foundational knowledge lessens anxiety associated with algorithmic opacity; (b) practical techniques enable proactive rather than mechanically passive system use; and (c) critical thinking preserves the students’ intellectual independence (Cetindamar et al., 2024; Kong et al., 2024; Long and Magerko, 2020; Ng et al., 2021). Consequently, AI literacy functions not only a technical competency but as a form of psychological resilience against the autonomy-broken tendencies of algorithmic personalization.

### 5.3 Theoretical contributions

This research makes several valuable theoretical contributions. First, it offers one of the earliest systematic longitudinal investigations of the personalization paradox, moving the field beyond conceptual conjecture to empirical evidence. Second, it extends control-value theory to AI-mediated educational contents, demonstrating that excellent perceived control in human-AI interaction not only encompasses control over learning processes, but also the unambiguous comprehension of system logic. Third, it emphasizes the primacy of emotional mechanisms over purely cognitive ones in explaining AI’s association with SRL, complementing prior research that has focused predominantly on both cognition and academic performance. Fourth, the conceptualization of AI literacy as a moderator introduces an important individual-difference dimension to the existing literature. Fifth, the integration of CVT and SRL theories in a single moderated mediation structure represents a innovative synthesis which advances both the two theoretical frameworks.

### 5.4 Practical implications

The findings offer actionable suggestions for multiple stakeholders:

*For educators*: They actively tutor students to utilize AI tools critically yet actively rather than passively yet mechanically. They incorporate reflective actions which help students distinguish when algorithmic recommendations may undermine their own planning program and monitoring tactics.

*For instructional designers and AI developers*: Adaptive systems can embed learner autonomy as a vital designing considering principles—for example, by allowing students to operate or adjust algorithmic recommendations. Therefore, they maintain a meaningful extension of agency. Meanwhile, their designs enhance system transparency by clearer explanations of recommendations’ rationales might foster knowledgeable control and trust.

*For university leaders and policymakers*: Integrate AI literacy education into vital curricula, treating it not only as a technical strategy but as an important component of digital citizenship and emotional well-establishment. They provide professional advances for faculty to support students’ vital engagement with AI tools.

*For future research*: Investigate the effectiveness of specific AI literacy interventions in weakened the personalization paradox, and develop cross-cultural variation in these effects.

### 5.5 Limitations and future directions

Several limitations warrant acknowledgement. First, the observational design, while incorporating temporal separation between measurement waves, precludes final causal inference. In the future, the similar research should employ randomized controlled trials or cross-lagged panel designs with baseline controls to establish causality. Second, reliance on self-reported measures introduces potential biases, future studies would benefit from integrating behavioral logs, eye-tracking data, or physiological indices. Third, the sample was restricted to Chinese university students limiting cross-cultural generalizability, particularly given that AI acceptance and trust may vary across cultural contexts. Fourth, the aggregation of diverse AI tools into a single composite measure may mask tool-specific effects; future research should implement multilevel analyses that distinguish among tool specifics. Fifth, common method bias, while assessed and found minimal, cannot be totally ruled out; multi-method designs could strengthen future researches.

### 5.6 Chapter summary

AI-driven adaptive learning environments represent a double-edged sword: while they offer unparalleled personalization, they simultaneously pose risks to university students’ self-regulated learning (SRL), mediated largely by negative changes in academic emotions. Fortunately, AI literacy becomes as a vital buffer against these adverse effects. As higher education increasingly embraces AI tools, it is imperative that the stakeholders balance technological efficiency with a genuine commitment to fostering learner autonomy and emotional well-being. Human-centered AI design and comprehensive AI literacy education are not optional enhancements—they are necessary safeguards to ensure that the promise of personalized learning does not come at the cost of students’ capacity for independence, self-directed learning.

## 6 Conclusion

This study investigated the personalization paradox in AI-adaptive learning environments by utilizing a three-wave longitudinal moderated mediation model leveraging control-value theory and SRL theory. The most important findings are threefold. First, acquired AI-adaptive environments were negatively associated with SRL, providing empirical support for the existence of the personalization paradox. Second, the relationship was quite significantly mediated by academic thinking and emotions—enjoyment, anxiety, and boredom—which totally accounted for over half of the entire effect. Third, AI literacy moderated the initial pathways from the AI environment to all these emotions, such that students with higher AI literacy went through attenuated negative emotional feedbacks. The theoretical contributions of this study are important: it offers empirical validation for the personalization paradox by using a rigorous longitudinal plan, extends CVT to AI-mediated learning contents, emphasizes emotional mechanisms alongside knowledgeable ones, and constructs AI literacy as a vital individual-difference moderator. Practically, the findings highlight the demand for educational institutions to embed AI literacy training into the entire curricula, for designers to construct transparent and autonomy-supportive adaptive systems, and for educators to positively scaffold students’ vital engagement with AI tools. These findings align with and extend former work by Koedinger et al. (2023) and Renz et al. (2020), moving beyond their knowledgeable accounts by confirming specific emotional mechanisms and a protective personal-difference factor.

Acknowledging the limitations of this report, observational data, and the Chinese researching sample, future research should apply experimental, cross-cultural, and multi-method designs to delve and refine these findings. Ultimately, the responsible integration of AI in higher education needs a entire approach that attends not merely to cognitive results but also to the emotional and autonomy-related directions of students’ future development.

## Data Availability

The original contributions presented in the study are included in the article/supplementary material, further inquiries can be directed to the corresponding author/s.
